# Cell and developmental biology: grand challenges

**DOI:** 10.3389/fcell.2024.1377073

**Published:** 2024-03-15

**Authors:** Amanda G. Fisher

**Affiliations:** Department of Biochemistry, University of Oxford, Oxford, United Kingdom

**Keywords:** chromosomes, organoids, comparative biology, atlas, methods, concepts

## Introducing a decade of progress

Cellular and developmental biology spans a huge portion of modern biology, so providing a brief synopsis of a decade of progress is a particularly daunting task. Collectively our interests range from a desire to understand how cells develop, replicate, interact, and age to more complex problems concerning how multicellular tissues form and the mechanisms required to ensure organismal-level physiological control and homeostasis throughout life. With this as a backdrop, highlighting just a handful of breakthroughs is challenging, so I apologise in advance for inevitable omissions in outlining some of the key areas where significant breakthroughs are helping to reshape the horizons of cell and developmental biology. I have structured this review to begin with genes, chromosomes, and protein-nucleic acid condensates, moving through to breakthrough technologies, and ending with a discussion of how these developments can revitalise comparative biology.

## Genome topology, chromatin, and loop extrusion

The linear arrangement of genes along chromosomes was initially mapped using genetics but was later confirmed and extended by high-throughput genome sequencing. Chromatin immunoprecipitation (ChIP) approaches have enabled us to map or “see” the binding of transcription factors and other protein complexes to specific loci (Park, 2009). Advanced microscopy and fluorescent *in situ* hybridisation (FISH), which provided a first glimpse of the spatial position of genes within the nucleus, has also been considerably scaled up in the last few years. However, it is the advent of specialised chromosome conformation capture technologies, dependent on physical proximity, that has enabled dimensional maps of genomic architecture to be established (Akgol Oksuz et al., 2021). In addition to providing robust quantitative measures of interactions between different parts of the genome, these contact maps have revealed topologically associated domains (TADs) and contact boundaries relevant for gene regulation in development (Rao et al., 2014). These studies are starting to decipher the architecture and grammar of genomes as well as revealing new phenomena, such as so-called jets or flares (Guo et al., 2022), where the direction of cis interaction (upstream or downstream of the point of study) is skewed to favour certain outcomes. Cohesin, a complex that was previously best known for holding together sister chromatids at mitosis (Gligoris et al., 2014), is heavily implicated in mediating cis-chromosome contacts of this sort, presumably by tethering chromatin loops and propagating directional loop extrusion (Guo et al., 2022; Zhang et al., 2023).

## Chromosome biology

The packaging of DNA into chromosomes as “thread-like structures that are visible as iconic X-shaped structures only during mitosis” has fascinated scientists for hundreds of years. However, efforts to determine the properties, folding, and higher-order structure of native chromosomes have been frustrated by the limited resolution offered by light microscopy. Single-particle cryogenic electron microscopy (CryoEM) can generate high-resolution structures of many biological assemblies (Berger et al., 2023), and as newer, cheaper, and more versatile instruments have become available (McMullan et al., 2023), these approaches together with focused ion beam (FIB) milling are now being used to elucidate native chromosome, chromatin, and nucleosome structures (Hou et al., 2023). Optical trapping approaches (or optical tweezers) can capture native chromosomes or DNA strands, allowing them to be manipulated and tested as experimental or biochemical polymers. Experiments of this kind are being used to resolve the mechanical properties of mitotic chromosomes and decipher the effects of removing key components, such as Topoisomerase 2 or cohesin (Djeghloul et al., 2020; Meijering et al., 2022). Over the next few years, it is very likely that significant progress will be made in refining the detailed ultrastructure of chromosomes as well as the condensation and de-condensation processes that accompany cell cycle progression, gene activation, and silencing.

## Biomolecular condensates and liquid–liquid phase separation

Biomolecular condensates are micron-scale compartments in eukaryotic cells that lack surrounding membranes, but nonetheless concentrate biomolecules, including proteins and nucleic acids. These structures have intrigued scientists for decades but until relatively recently were difficult to visualise and study (Banani et al., 2017). Concentrated non-stoichiometric assemblies of molecules can form when components change from a state of solubility and separate from the surrounding environment by phase transition (Hatters, 2023). It is now recognised that biomolecular condensate formation is critical for many biological processes in cell and developmental biology. These include well-characterised cytoplasmic, nuclear, and membrane functions, cellular stress and tissue-based immune responses, inflammation, and signalling (reviewed by Niu et al. (2023)), as well as epigenetic inheritance in worms (Brangwynne et al., 2009). Condensate formation is also an integral part of many disease processes and ongoing work focuses on using this new knowledge for therapeutic benefit (reviewed in Niu et al. (2023)). While there is still a lot to discover, progress in understanding how phase separation effectively ‘corrals’ important biomolecules within cells has radically changed our ideas on compartmentalisation and elevated the importance of self-organising molecular complexes.

## CRISPR/Cas9-based genome editing and protein degron technologies

A Nobel prize in Chemistry was awarded to Emmanuelle Charpentier and Jennifer Doudna for the discovery of so-called “genetic scissors” as a means to manipulate DNA and edit the genome of eukaryotes (Deltcheva et al., 2011; Jinek et al., 2012). The CRISPR-Cas9 system was initially described as an RNA-guided protective mechanism designed to protect prokaryotic genomes from infiltration by foreign mobile genetic elements. As a result of the modification of individual components of the CRISPR-Cas9 complex to enhance efficiency and broaden its application, this has become an incredibly versatile and powerful genome editing tool (reviewed by Gostimskaya, 2022) that is now in widespread use across the cell and developmental biology community, as well as in a clinical setting. Although CRISPR-Cas9-based approaches enable heritable changes to be engineered quickly in the genome of somatic and germline cells, for some experimental questions, acute protein depletion rather than genetic perturbation is required: for example, to test whether a specific protein is essential for a dynamic transition in differentiation, or to maintain identity at metaphase. For problems of this sort, the development of new proteolysis-targeting chimera (PROTACs) and inducible degron technologies is also proving invaluable (reviewed by de Wit and Nora (2023); Békés et al. (2022)).

## Single-cell RNA-seq, spatial profiling, and cell atlas projects

Until relatively recently, molecular biology was restricted to examining the characteristics of cellular populations *en masse,* meaning that cellular variation was diluted in favour of determining average gene expression. Although widely used, this approach was problematic when applied to minority cell populations, for example, cells at intermediate points along differentiation paths, or cells transiting stages of the cell cycle. Low-abundance cell types within populations were too often simply overlooked or misrepresented. Being able to discriminate gene expression (and translation) from an individual single cell using RNA-sequencing has transformed our knowledge of differentiation and tissue complexity (Tang et al., 2009; Chen et al., 2019; Cao et al., 2020; Satterlee et al., 2020), but also reinforced the need to understand inter-cell variability as a part of normal development, pathology, and disease progression (Jaitin et al., 2014; Rosenberg et al., 2018; VanInsberghe et al., 2021). With the advent of spatial profiling and its application to genomics, epigenomics, proteomics, and transcriptomics-based studies (Crosetto et al., 2015), reviewed by Marx (2021), there is an opportunity to launch several new cell atlas programs designed to provide reference cell maps of the entire body (Rozenblatt-Rosen et al., 2017; Cao et al., 2020; Haniffa et al., 2021). Once these are realised, they promise to provide comprehensive visual maps where gene expression, genetics, age, disease, and therapeutic response data can be overlaid, to help predict and treat a range of health conditions (Rood et al., 2022).

## Organoids and *ex vivo* tissue culture models

Over the last decade there has been a tremendous upsurge in the creation of new methods to study development *ex vivo* (reviewed by Corrò et al. (2020); Kim et al. (2020)). Driven by some of the pioneering work by Hans Clevers and others, it has become possible to ‘reconstitute’ the development of an array of different organs in man, and to investigate tissue regeneration, pathology, and drug responses in bespoke patient-specific organoids (Clevers, 2016; Driehuis et al., 2020). Detailed culture protocols are available to generate organoids representing many adult tissues, such as adult kidney, lung, gut, brain, and retina, as well as organoids developed from embryonic stem cells (or induced pluripotent stem cells, iPSCs) that can model early human gastrulation (Moris et al., 2020) or cortical brain development (Lancaster et al., 2013; Marsoner et al., 2018). The potential for progress in this area is vast, and current studies are enabling new global initiatives to be established, such as the organoid cell atlas that connects organoid and single-cell technologies (Bock et al., 2021). Longstanding questions remain as to how well *ex vivo* 3D cultures mimic the physiology of their *in vivo* counterparts, with the need to further integrate the impact of processes such as blood flow. It is, however, worth noting that *ex vivo* cellular models (neurospheres; gut and brain organoids) have been critical in establishing the tissue susceptibility of certain emerging human disease agents, including Zika virus, monkey pox, and severe acute respiratory syndrome (SARS)-coronavirus.

## Artificial intelligence, machine-based learning, and AlphaFold

Interdisciplinary research has flourished over the last few years, particularly at the intersection of computer science, mathematics, structural biology, statistics, genomics, and biology. Massive amounts of data generated from diverse sources, including clinical imaging, genome-wide sequencing, structural biology, epidemiology, and human Biobank projects, have offered an unrivalled opportunity to harness computer-based tools to draw out biological correlations and predictions. One of the best-known such tools reveals the 3D structure of proteins and was pioneered by the creators of Deepmind in collaboration with the EMBL-EBI. AlphaFold was launched in July 2022 and was the result of a 5-year endeavour to understand and predict protein structure across a range of organisms from humans to *E. coli*, yeast, fruit fly, mouse, and others (Jumper et al., 2021; Tunyasuvunakool et al., 2021; Varadi et al., 2022). Following on from this, it has become possible to predict the functional impacts of human missense variants (Cheng et al., 2023), something that is much needed to inform strategies to treat cancer and more generally, for human disease diagnostics. In the clinic, AI has the potential to transform medical imaging, diagnosis, and treatment (Oren et al., 2020) and, using data collected from wearable full-body motion suits, is already being used to monitor the progression of several genetically determined human diseases (Kadirvelu et al., 2023; Ricotti et al., 2023).

## Comparative biology

The consequences of rapid improvements in the speed, scale, and affordability of genomic sequencing, proteomics, and *ex-vivo* culture methods means that it is now possible to explore cells and development in a much wider range of plants, fungi, and animals than the narrow confines of available ‘model organisms’ previously used. A recent interesting example of this was provided by the Rowland laboratory, who have used 3D genomic comparisons of 24 eukaryotic species spread ‘across the tree of life’ to decipher the pivotal role of condensin II in genome architecture (Hoencamp et al., 2021). Comparative biology studies such as these are also useful in understanding more complex physiological processes, such as those that underpin sleep (Lakhiani et al., 2023) or hibernation (Willey and Korstanje, 2022), or are associated with long lifespans (for example, in the Bowhead whale, Keane et al., 2015). Such studies herald an exciting new era of comparative biology in which modern molecular, genomic, and computational tools can be harnessed to dissect interesting emergent properties that humans might wish to emulate. Although comparative biology was in the past sometimes pejoratively viewed as being “curiosity-led” or “purely academic,” on the contrary comparative biology is today offering fresh clues to an array of real-life problems, such as how to manage cancer risk with age (Vincze et al., 2022; Li et al., 2023) or prevent thrombosis upon immobilisation (Thienel et al., 2023).

## Trust in science

Despite a remarkable decade of progress in cell and developmental biology, the last few years have been marred by a sharp increase in cases of scientific misconduct and data falsification. This increase in cases contributes to an erosion of public trust in science, as well as confounding genuine scientific discovery. Fraudulent behaviour has unfortunately become more prevalent, including the use of paper mills (commercial enterprises that produce fraudulent manuscripts that resemble genuine scholarly articles), unethical referee networks, and the use of false or misleading email addresses to impersonate reputable scientific institutions. Frontiers employs a substantial number of staff within its research integrity office to combat this on our behalf and we are extremely grateful to them for their efforts. Setting this to one side, clearly many important pioneering discoveries have been made over the last decade in the fields of cell and developmental biology, and there is every indication that more are coming. For the next-generation of scientists who are working at this exciting frontier, there is so much to explore, enjoy, and discover.
